# Evaluation of access and utilization of EPI services amongst children 12-23 months in Kwahu Afram Plains, Eastern region, Ghana

**DOI:** 10.11604/pamj.2017.28.238.11538

**Published:** 2017-11-15

**Authors:** Emmanuel Tettey Sally, Ernest Kenu

**Affiliations:** 1 Department of Epidemiology and Disease Control, School of Public Health, University of Ghana, Accra, Ghana; 2 Eastern Regional Health Directorate, Ghana Health Service, Koforidua, Ghana

**Keywords:** Immunization Programs, Utilization, Program Accessibility, coverage, vaccination, EPI

## Abstract

**Introduction:**

High vaccination coverage is required to successfully control, eliminate and eradicate vaccine preventable diseases (VPDs). In Ghana, access complete vaccination coverage is 77%. However, sustaining high coverages in island communities such as Kwahu Afram Plains North (KAPN) is still a challenge.

**Methods:**

**Study site and settings**, an Island district. It targeted children aged 12–23 months. We used a modified WHO EPI 30 by 7 cluster sampling approach. Semi-structured questionnaires were employed for data collection. Wincosas and EpiInfo were used for data entry, management and analysis. The vaccination coverage, antigen-specific coverage calculated. The probability was set at 0.05 and the value was calculated to determine statistical significance of association.

**Results:**

Of the 480 records of children analysed, fully vaccinated accounted 81.3%, partially 16.7% and not vaccinated at all 2.1%. Access was 97.3% and utilization 91.2% with Pentavalent 1-3 dropout rate of 8.8%. Coverage for specific antigens were: BCG (97.1%), OPV 1/Pentavalent 1/PCV 1/Rotarix 1 (97.3%), OPV2/ Pentavalent 2/PCV 2/Rotarix 2 (94.0%), OPV3/ Pentavalent3/PCV 3 (88.8%), MR (87.7%) and YF (87.7%). Vaccination card availability, higher educational level of mothers and lower parity levels were significantly associated (p < 0.05) positively with childhood vaccination status. Invalid doses were 21.6% of childhood total vaccinations. Key reasons accounting for non-vaccination were: distant place of immunization 34.4 % (31/90), mother being busy 14.4% (13/90), vaccine unavailability 10.0% (9/90) and fears of side reactions 8.9% (8/90).

**Conclusion:**

EPI childhood vaccination coverage for January, 2016 in KAPN District was high. There is the need to focus on counteracting the reasons identified to account for vaccination failure. This would improve and sustain vaccination coverage.

## Introduction

Immunization remains one of the most important public health interventions and a cost effective strategy to reduce both the morbidity and mortality associated with infectious diseases. The World Health Organization, in 1974, initiated the Expanded Programme of Immunization (EPI) as a recommendation from the World Health Assembly (WHA). This was to help member states develop an immunization and surveillance programme against Measles, Poliomyelitis, Tuberculosis, Diphtheria, Pertussis and Tetanus. EPI has led to high vaccination coverages of about 80% for the six major vaccine preventable diseases: pertussis, childhood tuberculosis, tetanus, polio, measles and diphtheria [1]. WHO reports that an estimated number of 1.5 million deaths among children under-five years occurred in 2008 from diseases that could have been prevented through routine vaccinations. These diseases were measles, diphtheria, tuberculosis, tetanus, whooping cough/pertussis, poliomyelitis, yellow fever, hepatitis B and haemophilus influenza type B infection [2]. Immunization coverage levels and trends are used to monitor the performance of immunization services locally, nationally and internationally; to guide strategies for the eradication, elimination and control of vaccine-preventable diseases [2-4].

In developed countries, where accurate recording of immunization and reporting of diseases is in place, most vaccine-preventable diseases are at or near record lows [5]. About three quarters of the world’s child population is reached with the required vaccines, however in sub-Saharan Africa only half of the children get access to basic immunization. The worse happens in poor remote and hard to reach areas of developing countries, where one in twenty children have access to vaccination [6]. Globally, vaccination coverage is steady with 86% of children receiving DPT 3 in 2014 [7]. However, an estimated 18.7 million infants worldwide were not reached with basic vaccines in 2014. In Ghana, DPT3 administrative coverages were 93.2% and 89.9% respectively for 2014. The complete immunization coverage in Ghana in 1993 was 54.8% and this rose gradually in 2008 to 79.0% and declined to 77.3% in 2014 [8]. These unvaccinated children can build up as enough susceptible population over time and contribute to disease outbreaks. Ghana’s Expanded Programme on Immunization policy recommends that children receive Bacillus Calmette-Guerin (BCG) and Oral Polio Vaccine (OPV) at birth; three doses of Pentavalent vaccine, PCV and OPV at 6, 10 and 14 weeks of age, Rotarix at 6, 10 weeks of age and measles-rubella vaccine at 9 and 18 months of age [9]. Immunizations are recorded on child health records cards obtained from the clinics. In KAPN, DPT3 administrative coverage for 2014 was 89.9%. However, a complete vaccination coverage survey has not been conducted in KAPN within the past eight years. Administrative coverages, though with unreliable targets still records dropout of more than 10%. Reasons for this drop-out rate are still not known. Administrative data validation is a requirement by WHO. This study therefore sought to find out the coverage rate of children aged 12 - 23 months in KAPN as well as to identify the factors that influence it so as to propose recommendations for interventions and increase the immunization coverage.

## Methods

### Study site and settings

KAPN district was created in 1988 out of the Kwahu District Council as part of Local Government reform policy. The district is located in the northern- part of the Eastern Region, covering a total land mass of 3,210km2. The district capital is Donkorkrom with 238 communities. It may be described as more of an island than peninsular as it can only be reached by ferries in the South-west and North-east. The districts estimated population is 113,806. It is a rural district; majority are farmers with low socio-economic status and health service access far below standard.

Community-based Health Planning and Services (CHPS) centres form majority of the health facilities in the district. Routine vaccinations activities are organized using two main approaches, thus static and outreach. All CHPS centres render static vaccinations services every day. However, each facility has a specific day in a week where Child Welfare Clinic (CWC) are organized; usually linked to their market days. There are also planned days for outreach services at the community level where they are supported by community health volunteers. These services availability is solely dependent on resources (staff, funds, fuel, EPI logistics and boats, motor-bikes). The session of vaccinations begins with a health talk on a subject by a nurse. All vaccinations are provided free of charge.

### Study design

A community-based cross-sectional study was conducted in which 480 mothers/guardians with children aged 12 - 23 months were interviewed. The participants had to meet the inclusion criteria of being residents in the study area for a period of not less than 1 year. A two-stage cluster sampling was used. Stage one, cluster identification was done through the use of Ghana statistical service department Electoral Areas (EA) as clusters. Each EA was considered as clusters, which represented the primary sampling unit (PSU). In all thirty (30) clusters were selected randomly. No village was excluded from the sampling frame. Maps of the selected clusters were obtained from the KAPN health directorate which aided in the description of the boundaries, using local people. The entire district is considered as rural, hence there was no need to stratify by urban and rural. Stage two, was the selection of households in the various clusters. Households were selected randomly. Eligible children were selected from households until the required number of sixteen was met for the cluster. Where there were two eligible children/twins, the younger one was chosen.

A pre-tested semi structured questionnaire was used in data collection. Information collected included the socio-demographic characteristics, vaccination status of the children and other factors hindering full vaccinations of children. Child health cards were used to support in the assessment of the vaccination status of the children. In case of absence of the child health cards, mothers/caretakers of the respective children were asked to recall the immunization history guided by the count of vaccinations received in each site as provided for in the national schedule. BCG coverage was also verified by physically checking the vaccination site of all surveyed children for the presence of a BCG scar.

### Ethical approval

Institutional Review Board for Noguchi Memorial Institute for Medical Research, gave approval for the study. Permission was also sought from the District Assembly and Health Directorate. Prior consent was obtained in accordance with the ethical guidelines. All stake holders were informed about the study. Consent was sought from caregivers of selected children for the study.

### Data management and analysis

The data from the field was cleaned, coded and double entered into WinCosas and EpiInfo statistical package. The data were analyzed and descriptive statistics was run for continues variables. The vaccination coverage, antigen-specific coverage calculated and confidence interval of 95% determined. Vaccination coverages were calculated as crude, valid before 52 weeks. Quality of the vaccination was accessed by the presence of vaccination cards and invalid doses given. Bivariate analysis using Pearson’s Chi -Square test was used to evaluate the association between the vaccination status and socio-demographic factors like sex of child, vaccination card availability, mother’s age, parity, educational status, marital status, occupation and religious affiliation. The probability was set at 0.05 and the value was calculated to determine statistical significance of association. Reasons why targeted children were not vaccinated was solicited from caregivers of children whose records indicated were either partially or not vaccinated at all. The various factors were analysed as either lack of information, lack of motivation and/or obstacles. Microsoft Office Excel 2016 was used to generate tables and figures.

## Results

### General characteristics of the study participants

Almost half of the care givers who participants were 47.1% (226/480), were aged between 25 and 34 years and the mean age was 28.6±6.9. Females were more 96.9% (465/480) than the males 3.1% (15/480). Twenty six percent 26.0% (125/480) had no formal education, most 33.3% (160/480) had attained secondary school education (Junior) and few 3.1% (15/480) reaching tertiary education. Majority of participants 26.9% (129/480) reported to have five or more children. Amongst children surveyed, 50.4% (242/480) were females and 49.6% (238/480) were males (Table 1).

**Table 1:** Socio-demographic characteristics of surveyed mothers/caregivers

Characteristics of surveyed Mothers/Caregiver	Caregiver surveyed for child immunization	
	*Number (n=480)*	*Percentage (%)*
**Sex**		
Male	15	3.1
Female	465	96.9
**Age**		
15 – 24	147	30.6
25 – 34	226	47.1
35 – 44	102	21.3
≥ 45	5	1.0
**Parity**		
1 Child	94	19.6
2 Children	105	21.9
3 Children	74	15.4
4 Children	78	16.4
≥ 5 Children	129	26.9
**Marital status**		
Single	75	15.6
Married	392	81.7
Divorced	3	0.7
Separated	5	1.0
Widowed	5	1.0
**Educational status/level**		
No Formal Education	125	26.0
Primary	139	29.0
JHS+/MSLC+	160	33.3
SHS/Tech/Voc	41	8.5
Tertiary	15	3.1
**Occupation**		
Unemployed	73	15.2
Farmer/Artisan	204	42.5
Trader/Self employed	178	37.1
Student	11	2.3
Salary Earner	14	2.9
**Religious affiliation**		
Christian	441	91.9
Muslim	26	5.4
Traditionalist	13	2.7

+ **JHS:** Junior High School

+ **MSLC:** Middle School Leaving Certificate

### The immunization coverage in KAPN

The study showed that 390 (81.3%) of the children were fully immunized by card and history. Percentage coverages for all the antigens were more than 80%. Measles-rubella and yellow fever vaccines recorded the minimum of 87.7% (95% CI: 74.3 - 101.1) with OPV 1 and Pentavalent 1 recording the maximum of 97.3% (95% CI: 90.7 - 103.9). The BCG scar rate was 93.0 %. The rate however, among those vaccinated by card only was 91.9%. The drop-out rate for Penta1-3 and BCG-MR were 8.8% and 9.7% respectively. Card availability was 91.7% (440/480). The survey findings showed that invalid doses accounted for up to 21.6% (883 out of 6569) of childhood vaccinations (Table 2).

**Table 2:** Vaccination coverage (crude and valid) by antigen among 12 - 23 months

Doses/Antigen/FVC	Crude coverage				Valid coverage		Valid coverage ≤ 52 weeks	
	Card + History	%	Card	%	Card	%	Card	%
**BCG Vaccine**								
BCG	466	97.1	421	87.7	422	87.9	421	87.7
BCG							228+	47.5
**Oral Polio Vaccine**								
OPV 0	311	64.8	268	55.8	268	55.8	208+	43.3
OPV 1	467	97.3	430	89.6	387	80.6	387	80.6
OPV 2	451	94.0	417	86.9	342	79.4	342	71.3
OPV 3	426	88.8	395	82.3	305	63.5	305	63.5
**Pentavalent vaccine**								
Pentavalent1	467	97.3	430	89.6	387	80.6	387	80.6
Pentavalent2	451	94.0	417	86.9	342	79.4	342	71.3
Pentavalent3	426	88.8	395	82.3	305	63.5	305	63.5
**PCV 13**								
PCV 1	467	97.3	430	89.6	387	80.6	387	80.6
PCV 2	451	94.0	417	86.9	342	79.4	342	71.3
PCV 3	426	88.8	395	82.3	305	63.5	305	63.5
**Yellow Fever Vaccine**								
YF	421	87.7	387	80.6	316	65.8	272	56.7
**MR**								
MR	421	87.7	387	80.6	316	65.8	272	56.7
**Rotavirus vaccine**								
Rotarix 1	467	97.3	430	89.6	387	80.6	387	80.6
Rotarix 2	451	94.0	417	86.9	342	79.4	342	71.3
**FVC**								
Fully Vacc. Child	390	81.3	355	74.0	226	47.1	201	41.9

**BCG:** Bacillus Calmette – Guerin vaccine, **PCV:** Pneumococcal Conjugate Vaccine, **FVC:** Fully Vaccinated Child, **MR:**Measles-Rubella vaccine, ^+^Immunization given at birth, thus within 2 weeks after birth

### Socio-demographic characteristics of study participants and immunization coverage

Some factors that were found to be significantly associated with immunization coverage include: level of education (p < 0.001), number of children within the family (p < 0.008) and vaccination card availability (p = 0.001) (Table 3).

**Table 3:** Socio-demographic factors associated with childhood immunization status

Characteristics of surveyed Mothers/Caregiver & child	Total (n=480)	Vaccination status		*X2 (p-value)^1^*
		*Fully – n (%)*	*Not Fully - n (%)*	
**Sex of Child**				1.27 (0.260)
Male	239	199 (41.5)	40 (8.3)	
Female	241	191 (39.8)	50 (10.4)	
**Age of mother (years)**				3.75 (0.290)
15 – 24	147	115 (24.0)	32 (6.7)	
25 – 34	226	190 (39.6)	36 (7.5)	
35 – 44	102	80 (16.7)	22 (4.6)	
≥ 45	5	5 (1)	0 (0)	
**Parity**				9.74 **(0.008)**
1 Child (Nulliparous)	94	79 (16.5)	15 (3.1)	
2 – 4 Children (Multiparous)	257	218 (45.4)	39 (8.1)	
≥ 5 Children (Grand multiparous)	129	93 (19.4)	36 (7.5)	
**Marital status**				0.206 (0.650)
Married	392	317 (66.0)	75 (15.6)	
Not married	88	73 (15.2)	15 (3.1)	
**Educational status/level**				25.0 **(0.001)**
No Formal Education	125	84 (17.5)	41 (8.5)	
Primary	139	118 (24.6)	21 (4.3)	
JHS/MSLC	160	140 (29.2)	20 (4.2)	
SHS/Tech/Voc	41	33 (6.9)	8 (1.7)	
Tertiary	15	15 (3.1)	0 (0.0)	
**Occupation**				0.716 (0.397)
Unemployed	84	71 (14.8)	13 (2.7)	
Employed	396	319 (66.5)	77 (16.0)	
**Religious affiliation**				0.522 (0.470)
Christian	441	360 (75)	81 (16.9)	
Others	39	30 (6.25)	9 (1.9)	
**Vaccination card**				23.7 **(0.001)**
Available	440	369 (76.9)	71 (14.8)	
Not Available	40	21 (4.3)	19 (4.0)	

### Reasons for incomplete vaccinations

**Figure 1:**
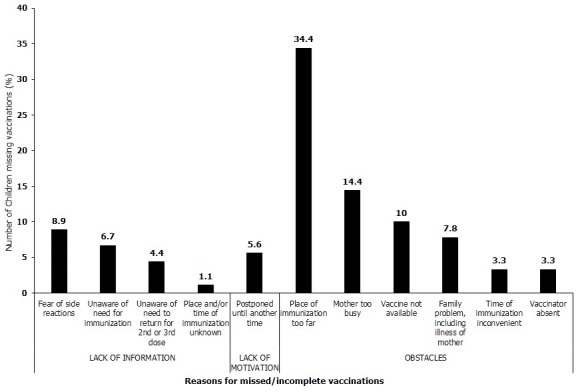
Specific reasons for missed/incomplete vaccinations among children 12-23 months (N = 90)

The participants who could not complete (partially and not vaccinated) the routine childhood immunization schedule for their children were asked for their reasons for the vaccination failure. These reasons were grouped as: a) lack of information; b) lack of motivation and c) obstacles. The survey noted that majority 73.3% (66/90) of reasons for non-vaccination were due to obstacles. Details are shown in Figure 1.

## Discussion

The study evaluated access and utilization of EPI services in the KAPN district of Eastern region. It determined vaccination coverage of children aged 12 - 23 months, quality of the vaccinations provided and reasons why eligible children were not vaccinated. Crude coverage for fully, partially and not vaccinated children were 81.3% (390/480), 16.7% (80/480) and 2.1% (10 of 480) respectively. Reducing the number of children who had never been vaccinated is crucial. The hard-to-reach populations in KAPN must be provided with additional resources to cope with logistical, transport, cold chain and human resources limitations. Contrary to this study, Antai [10] found childhood vaccination coverage uptake in Nigeria for full vaccination to be 13.5%. They attributed this low coverage to individual and community level disparities affecting immunization.

Coverages by antigen were generally high: BCG, Pentavalent 1, Pentavalent 3 and measles/rubella were 97.1% (466/480), 97.3% (467/480), 88.8% (426/480) and 87.7% (421/480) respectively. These high coverages reported are again supported by Osei-Sarpong’s work on “Factors associated with vaccination status of children in the Ga South Municipality, 2014” [11]. Another study conducted in Gondar, Ethiopia among 12 - 24 months old children reported 47.4% as fully vaccinated [12]. However, administrative coverage reported by the KAPN for BCG, Pentavalent 1, Pentavalent 3 and measles/rubella were 88.1%, 92.5%, 79.9% and 76.6% respectively [13]. Generally, these were low compared with this study. Again, the vaccination coverages were found in this study to be quite lower in sub-districts and communities that are “islands of an island”; it could be due to difficulty in rendering health care services at this area since geographical access is clearly bad with limited network.

Although KAPN has a difficult terrain and made up several island communities, the study reported good access for childhood immunizations. This was proven by the high coverage of Pentavalent 1 (97.1%). Similar findings were observed in different settings by Amanya [14] and Osei-Sarpong, 2014 [11]. Administrative coverage for 2014, was good (92.5%) but slightly lower than study findings. In general, Ghana’s EPI has gained success in the area of access. As a country access coverage contradicts Wolfson et al.’s report which indicated that for developing countries only one in twenty children have access to vaccinations [6]. It must however, be noted that KAPN district’s high coverage masks the poor performance of some communities and sub-districts.

A key issue that needs attention is the utilization of EPI services. Caregivers do not report to complete vaccination schedules; example Pentavalent drop out was 8.8%. Poor utilization (high drop outs) noticed in the study is supported by KAPN’s 2014 administrative data. To reduce dropout rates appointments should not only be written for mothers but in addition once any mother defaults on any of the contact vaccines, defaulter tracing in the form of home visit should be instituted. This approach will help strengthen the delivery of static immunization services [15]. On vaccination quality, card retention rate for childhood vaccinations (91.7%) were good. These childhood vaccination rates were higher than the rates recorded by the Ghana Demographic and Health Survey, 2014 (88.2%) [8]. Variables found to be significantly associated with vaccination status (crude) of children aged 12-23 months in KAPN districts were vaccination card availability, mother’s educational level and parity. The findings are similar to outcome of the work done by Osei-Sarpong in Ga South, 2014 [11]. This implies efforts channelled towards these factors may possibly improve childhood vaccination status.

Some vaccinations provided to children 21.6% (883/6569) surveyed were invalid. This could mainly be due to inappropriate screening of target groups by vaccinators prior to vaccinations and/or wrong documentation. In other to achieve maximum benefits for vaccinations all vaccines must be delivered meeting the basic validity criteria.

A total of 90 (18.8%) children surveyed were not fully vaccinated. This was largely because place of immunization was too far (35.6%), mother was too busy (14.9%), vaccine not available (10.0%) and fears of side reactions (9.2%). These findings support several results of various studies [14, 16-18]. Family problems including illness of mother, unaware of the need for vaccination, unaware of the need to return for 2nd and 3rddoses, postponement until another time, vaccinator absent and timing of vaccination inconvenient also recorded percentages ranging from 5 to 10. KAPN district is sparsely populated especially on the “islands of island” and outreach points may indeed be far from mothers as identified. Vaccines supply during 2014 in the district was erratic hence the finding [12]. This study was prone to recall bias since the respondents, who did not have the child health cards, were asked to recall the vaccines that were administered to their children, 1 - 11 months earlier.

## Conclusion

EPI childhood vaccination coverage in KAPN is high. Vaccination card availability, mother’s educational level and parity were significantly associated with childhood vaccination status. EPI service quality, card retention rate for childhood vaccinations was good. Total invalid doses administered to children accounted for 21.6%. Main reasons accounting for children 12 - 23 months not been vaccinated were: place of immunization too far, mother too busy and vaccine not available. Efforts to improve and sustain vaccination coverage should consider associated factors found in this study. There is also the need to focus on counteracting the reasons identified to account for incomplete vaccination. Again, to achieve optimal public health benefits, the study recommends that dropout rates are reduced through reliable vaccine supply, in-service trainings for health workers, increasing outreach points, using behaviour-change-communications strategies, client education on possible side effects/number of schedules/need to complete schedule and implementing systems for defaulter prevention and tracing.

### What is known about this topic

- General high vaccination coverage for childhood vaccinations (administrative).

### What this study adds

- About one out of four children vaccinated receives an invalid dose.

## Competing interests

The authors declare no competing interests.
